# Assessments of bilateral asymmetry with application in human skull analysis

**DOI:** 10.1371/journal.pone.0258146

**Published:** 2021-10-06

**Authors:** M. Hou, M. J. Fagan

**Affiliations:** Department of Engineering, Medical and Biological Engineering, Faculty of Science & Engineering, University of Hull, Hull, United Kingdom; Institute of Evolutionary Biology, Pompeu Fabra University, SPAIN

## Abstract

As a common feature, bilateral symmetry of biological forms is ubiquitous, but in fact rarely exact. In a setting of analytic geometry, bilateral symmetry is defined with respect to a point, line or plane, and the well-known notions of fluctuating asymmetry, directional asymmetry and antisymmetry are recast. A meticulous scheme for asymmetry assessments is proposed and explicit solutions to them are derived. An investigation into observational errors of points representing the geometric structure of an object offers a baseline reference for asymmetry assessment of the object. The proposed assessments are applicable to individual, part or all point pairs at both individual and collective levels. The exact relationship between the developed treatments and the widely used Procrustes method in asymmetry assessment is examined. An application of the proposed assessments to a large collection of human skull data in the form of 3D landmark coordinates finds: (a) asymmetry of most skulls is not fluctuating, but directional if measured about a plane fitted to shared landmarks or side landmarks for balancing; (b) asymmetry becomes completely fluctuating if one side of a skull could be slightly rotated and translated with respect to the other side; (c) female skulls are more asymmetric than male skulls. The methodology developed in this study is rigorous and transparent, and lays an analytical base for investigation of structural symmetries and asymmetries in a wide range of biological and medical applications.

## Introduction

Often the geometric structure of a biological object is represented by a collection of anatomical landmarks divided into three subsets: two of them contain the paired landmarks on two sides of an object respectively, while the third includes the landmarks shared by both sides. The two sides of the object are typically assumed to be geometrically symmetric about a plane in the closest proximity to the shared landmarks. Symmetry can take different forms and abnormal biological development may be reflected in noticeable structural asymmetries. As a fundamental notion, bilateral symmetry has been an important topic in morphometrics, e.g. [1], while other forms of symmetry have been found [2] traceable on the basis of bilateral symmetry. Investigation of bilateral asymmetry is important in biological studies for assessing developmental variability and instability, and also useful in clinical and surgical applications for diagnosing abnormalities and alignment of limbs and organs.

Due to bioecological variations such as genetic mutations and environmental changes together with observational errors caused by uncertainties in landmark placements and measurements, practically no biological object is exactly bilaterally symmetric. The sufficiency of one-side-only analysis of bilaterally symmetric objects was questioned and methods for use or recovery of missing information with two-side data were suggested [3]. In biological studies different ideas of asymmetry assessment have been used to characterise deviations from bilateral symmetry. The three most widely used notions are fluctuating asymmetry (small, random deviations), directional asymmetry (a consistent bias toward a given side) and antisymmetry (a consistent bias toward a random side) [1, 4]. Long standing research in seemingly simple fluctuating asymmetry has been fruitful but the topic remains controversial in particular when used to assess developmental instability [5–9]. The continuing debate demonstrates a lack of certainty about real connections between often subtle variations in the morphological and biological phenomena. Most studies have focused on fluctuating asymmetry as a measure of developmental instability, but many conflicting results may actually be due to inaccuracies in its assessment [4, 8]. Hence, the current study attempts to refine the three asymmetry notions by recasting them in the framework of analytic geometry to facilitate more reliable and accurate asymmetry assessments. The development of a rigorous analytical methodology for asymmetry assessments benefits the evaluation of developmental instability and the diagnosis of diseases causing structural deviations in biological objects.

Traditional approaches [1, 10] use a number of length measurements of the two-side structure of an object to investigate bilateral symmetry. While these measurements capture some morphometric traits in a population, they do not offer a direct assessment of the deviations of individual objects from bilateral symmetry. A more reliable but less effective method appears to be the Euclidean distance matrix analysis (EDMA) [11]. It was adopted in a study of human skull asymmetry [12], where all linear distance differences between two-side landmarks to middle landmarks of a skull were used in the analysis. The full set of linear distances has however a high level of redundancy [13] and only indirectly indicates 3D asymmetry. More systematic studies, e.g. [14, 15], consider morphometric traits collectively, and use a sophisticated combination of the two methods: Procrustes superimposition and statistical analysis in a software tool [16]. The former is about object alignment after reflection of anatomical landmarks on one object side about a prespecified reference, while the latter provides a statistical means of assessing degrees of asymmetry differences between the reflected landmarks and the original ones on the other side. Such a combination has been used in many applications to assess, for instance, human skulls [17] and femurs [18]. Using a mixed method of the Procrustes landmark registration and traditional left-right trait calculations, a negative correlation of bodily fluctuating asymmetry has been established with bodily attractiveness [19].

The current study examines bilateral asymmetry directly in an analytic geometry setting and derives explicit solutions for several types of asymmetry assessments. These types are naturally linked to assessment of the two known categories: object symmetry and matching symmetry [14, 20], meaning symmetry of two inseparable parts, and that of two separate parts of an object, respectively. The proposed asymmetry assessments are applicable to individual landmark pairs and objects, or all of them as a whole in a population. The direct treatment allows evaluation of bilateral asymmetry at both individual and collective levels and determination of its nature (fluctuating asymmetry, directional asymmetry or antisymmetry) in a population. The exact relationship of the proposed asymmetry assessments with the widely used Procrustes method is examined. An application of the developed method in this study to a large collection of human skulls provides some quantitative insights into the nature and extent to which human skulls are bilaterally asymmetric under influence of observational errors of skull landmarks.

## Materials and methods

### Notations

Set {*x*_*i*_} collects a given number of scalars or (column) vectors. Symbol ′ stands for the transpose of a vector or matrix, and ‘det’ and ‘tr’ abbreviate the determinate and trace of a square matrix respectively. |*a*| is the absolute value of scalar *a*, and $\left\|A\right\|=\sqrt{\text{tr}(A^{\prime }A)}$ is the norm of vector or matrix *A*. The diagonal matrix with *n* scalars {*a*_*i*_} is denoted by *diag*(*a*_1_, …, *a*_*n*_), and becomes the identity matrix, denoted by *I*, when *a*_*i*_ = 1 for all *i*. In singular value decomposition *A* = *U*Λ*V*′, diagonal matrix Λ consists of the singular values, and the columns of matrices *U* and *V* contain the left- and right-singular vectors of *A* respectively. Denoted by *a* × *b* is the cross product of vectors *a* and *b*. With *sgn*(0) = 0, *sgn*(*x*) = *x*/|*x*| for scalar *x* ≠ 0 is the sign function. Denoted by $x∼N(\mu_{x},\sigma_{x}^{2})$ is a random variable following the normal distribution with mean *μ*_*x*_ and standard deviation *σ*_*x*_, similarly $x∼N(\bar{x},\Sigma_{x})$ indicates a normal random vector with mean $\bar{x}$ and variance matrix Σ_*x*_. *A*^+^ is the Moore-Penrose inverse of matrix *A*. *Pr*(*A*) is the probability of event *A*. All quantities under consideration are of real numbers.

### Definitions of symmetry

**Fig 1:**
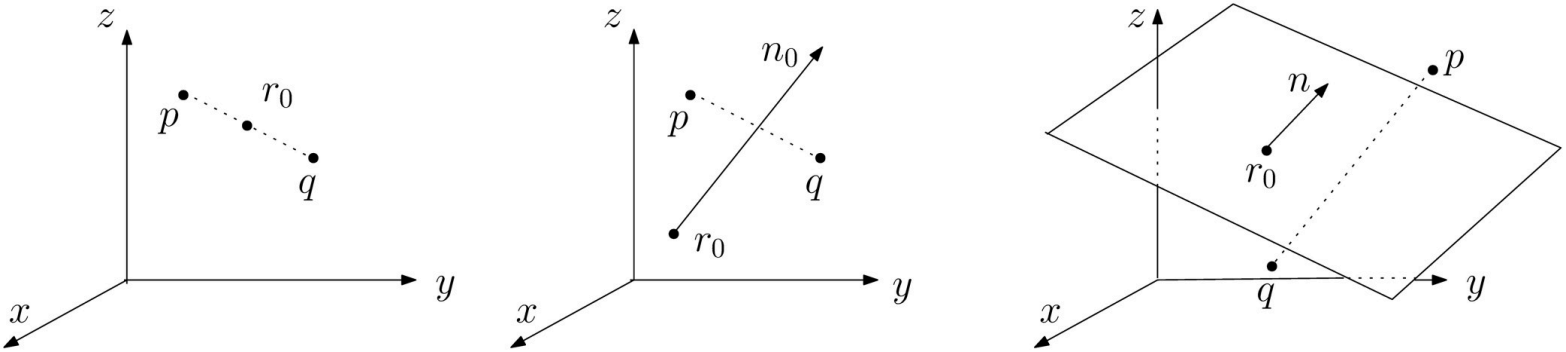
Point, line and plane reflections between 3D points *p* and *q*.

Explicitly or implicitly symmetry always has a reference. Fig 1 illustrates reflection symmetries of a pair of points (*p*, *q*) about a point *r*_0_, a line (*n*_0_, *r*_0_) and a plane (*n*, *d*), called *midpoint*, *midline* and *midplane* respectively, in a 3D space. Here, *n*_0_ is the direction vector of the line passing *r*_0_, *n* the normal to the plane, and *d* the (signed perpendicular) distance to the plane from the origin of a reference frame which is a 3D Cartesian coordinate system. Both *n*_0_ and *n* are unit vectors.

Respectively on to references *r*_0_, (*n*_0_, *r*_0_) and (*n*, *d*), the (orthogonal) *projections* of point *p* are *r*_0_, *p* + *I*_0_(*r*_0_ − *p*) and *p* + (*d* − *n*′*p*)*n* with $I_{0}=I-n_{0}n_{0}^{\prime }$.

**Definition 1** *A pair of points* (*p*, *q*) *is said to be symmetric about a reference if q reflects p (or vice versa) through the reference, namely if*

$$\begin{array}{c} q=\{\begin{array}{cc} p+2(r_{0}-p), & for\;point\;symmetry;\\ p+2I_{0}\left(r_{0}-p\right), & for\;line\;symmetry;\\ p+2(d-n^{\prime }p)n, & for\;plane\;symmetry. \end{array} \end{array}$$ (1)

**Definition 2** *Sets* {*p*_*i*_} *and* {*q*_*i*_} *with the same number of elements are said to be symmetric about a reference if pair* (*p*_*i*_, *q*_*i*_) *for all i is so*.

**Definition 3** *Sets* {*p*_*i*_} *and* {*q*_*i*_} *with the same number of elements are said to be symmetric about a reference by transformation* (*a*, *R*, *t*) *(scaling, rotation and translation) on* {*q*_*i*_} *if pair* (*p*_*i*_, *aRq*_*i*_ + *t*) *for all* *i* *is symmetric about the reference*.

To be linked to symmetry notions used in biological applications, the point, line and plane symmetries are associated with spherical, cylindrical and mirror symmetries respectively, while symmetry by transformation generally covers scaling, radial and glide symmetries. The three actions of a transformation do not have to be applied altogether. For instance, (*a*, *I*, 0) means no translation or rotation, but scaling, while (1, *R*, 0) indicates rotation only.

### Types of asymmetry assessment

Consider an object having {*p*_*i*_} and {*q*_*i*_} respectively on its two sides, and {*r*_*i*_} shared by both sides. Each of {*p*_*i*_} and {*q*_*i*_} consists of *m* points, and {*r*_*i*_} *k* points, with integers *m* > 0 and *k* ≥ 0. Hence, an object under consideration has at least one point on each side, and some or possibly no point shared by both sides.

#### Asymmetry index

Due to structural deviations and observational errors in an object, inevitably none of the relationships in (1) can hold exactly in real applications. The deviation of (*p*_*i*_, *q*_*i*_) from exact symmetry, called the *asymmetry index*, is defined by, for *i* = 1, 2, …, *m*,

$$\begin{array}{c} e_{i}=\{\begin{array}{cc} q_{i}-p_{i}-2\left(r_{0}-p_{i}\right), & \text{for}\;\text{point}\;\text{asymmetry};\\ q_{i}-p_{i}-2I_{0}\left(r_{0}-p_{i}\right), & \text{for}\;\text{line}\;\text{asymmetry};\\ q_{i}-p_{i}-2\left(d-n^{\prime }p_{i}\right)n, & \text{for}\;\text{plane}\;\text{asymmetry}. \end{array} \end{array}$$ (2)

When transformation (*a*, *R*, *t*) is applied to *q*_*i*_ to have *aRq*_*i*_ + *t*, the corresponding update of *e*_*i*_ in (2) is denoted by $e_{i}^{a}$. Indices {*e*_*i*_} and $\left\{e_{i}^{a}\right\}$ can be used for asymmetry assessments at individual and collective levels.

#### Assessment types

By least squares of asymmetry indices {*e*_*i*_} or $\left\{e_{i}^{a}\right\}$, a midpoint *r*_0_, midline (*n*_0_, *r*_0_) or midplane (*n*, *d*) as a symmetry reference and a transformation (*a*, *R*, *t*) of points on one side of the reference, say {*q*_*i*_}, can be determined, leading to different types of asymmetry assessments.

Type 0 (middle fitting): A reference is determined by best fitting to {*r*_*i*_}.
Type 1 (side balancing): A reference is determined by best balancing {*p*_*i*_} and {*q*_*i*_} on its two sides.
Type *α* (mixed types 0 and 1): A reference is determined by simultaneously fitting to {*r*_*i*_} and best balancing {*p*_*i*_} and {*q*_*i*_} on its two sides, with weightings 1 − *α* and *α* respectively, for 0 ≤ *α* ≤ 1.
Type 2 (side transforming): A general transformation (*a*, *R*, *t*) of {*q*_*i*_} is determined by making {*p*_*i*_} and {*aRq*_*i*_ + *t*} most symmetric about a given reference.

The least squares criteria for determination of these references and transformations with respect to the four assessment types are

$$\begin{array}{c} J_{0}=\sum_{i=1}^{k}\delta_{i}^{2},\;J_{1}=\sum_{i=1}^{m}\|e_{i}{\|}^{2},\;J_{\alpha }=\left(1-\alpha \right)J_{0}+\alpha J_{1},\;J_{2}=\sum_{i=1}^{m}{\left\|e_{i}^{a}\right\|}^{2}, \end{array}$$ (3)

where *δ*_*i*_ stands for the perpendicular distances of point *r*_*i*_ to these references:

$$\begin{array}{c} \delta_{i}=\{\begin{array}{cc} \|r_{0}-r_{i}\|, & \text{for}\;\text{point}\;\text{asymmetry;}\\ \|I_{0}\left(r_{0}-r_{i}\right)\|, & \text{for}\;\text{line}\;\text{asymmetry;}\\ |d-n^{\prime }r_{i}|, & \text{for}\;\text{plane}\;\text{asymmetry.} \end{array} \end{array}$$ (4)

Clearly, types 0 and 1 are special cases of type *α*, and in the case of no transformation, namely (*a*, *R*, *t*) = (1, *I*, 0), type 2 is reduced back to type 0, 1 or *α* depending on which of the three references is considered. It is possible to further combine types *α* and 2 to form just one general assessment type by considering the criterion *J*_*β*_ = (1 − *β*)*J*_*α*_ + *βJ*_2_ with 0 ≤ *β* ≤ 1. Having such a universal type would be theoretically attractive and effective for algorithm implementation, but is not pursued for sake of clarity and accessibility of the key methodology developed in this study.

#### Specifications of references and transformations

In terms of a few data vectors, centroids and matrices defined as

$$\begin{array}{ccc} & & r=\frac{1}{k}\sum_{i=1}^{k}r_{i},\;p=\frac{1}{m}\sum_{i=1}^{m}p_{i},\;q=\frac{1}{m}\sum_{i=1}^{m}q_{i}, \end{array}$$ (5)

$$\begin{array}{ccc} & & r_{\alpha }=\frac{\left(1-\alpha )kr+2\alpha m(q+p\right)}{(1-\alpha )k+4\alpha m}, \end{array}$$ (6)

$$\begin{array}{ccc} & & R_{\alpha }={\left[\begin{array}{cccc} r_{1}-r_{\alpha }, & r_{2}-r_{\alpha }, & \cdots , & r_{k}-r_{\alpha } \end{array}\right]}^{\prime }, \end{array}$$ (7)

$$\begin{array}{ccc} & & P_{\alpha }={\left[\begin{array}{cccc} p_{1}-r_{\alpha }, & p_{2}-r_{\alpha }, & \cdots , & p_{m}-r_{\alpha } \end{array}\right]}^{\prime }, \end{array}$$ (8)

$$\begin{array}{ccc} & & Q_{\alpha }={\left[\begin{array}{cccc} q_{1}-r_{\alpha }, & q_{2}-r_{\alpha }, & \cdots , & q_{m}-r_{\alpha } \end{array}\right]}^{\prime }, \end{array}$$ (9)

$$\begin{array}{ccc} & & X_{\alpha }=\left(1-\alpha \right)R_{\alpha }^{\prime }R_{\alpha }+2\alpha \left(P_{\alpha }^{\prime }Q_{\alpha }+Q_{\alpha }^{\prime }P_{\alpha }\right), \end{array}$$ (10)

$$\begin{array}{ccc} & & P={\left[\begin{array}{cccc} p_{1}-p, & p_{2}-p, & \cdots , & p_{m}-p \end{array}\right]}^{\prime }, \end{array}$$ (11)

$$\begin{array}{ccc} & & Q={\left[\begin{array}{cccc} q_{1}-q, & q_{2}-q, & \cdots , & q_{m}-q \end{array}\right]}^{\prime }, \end{array}$$ (12)

$$\begin{array}{ccc} & & f_{i}=p_{i}+2I_{0}\left(r_{0}-p_{i}\right),\;g_{i}=p_{i}+2\left(d-n^{\prime }p_{i}\right)n, \end{array}$$ (13)

$$\begin{array}{ccc} & & f=\frac{1}{m}\sum_{i=1}^{m}f_{i},\;g=\frac{1}{m}\sum_{i=1}^{m}g_{i}, \end{array}$$ (14)

$$\begin{array}{ccc} & & F={\left[\begin{array}{cccc} f_{1}-f, & f_{2}-f, & \cdots , & f_{m}-f \end{array}\right]}^{\prime }, \end{array}$$ (15)

$$\begin{array}{ccc} & & G={\left[\begin{array}{cccc} g_{1}-g, & g_{2}-g, & \cdots , & g_{m}-g \end{array}\right]}^{\prime }, \end{array}$$ (16)

references and transformations for assessment types *α* and 2 are specified as follows with verifications given in S1 Appendix, and types 0 and 1 specified through type *α*.

The references for type *α* assessment with 0 ≤ *α* ≤ 1 are

$$\begin{array}{c} \left\{ \begin{array}{cc} r_{0}=r_{\alpha }, & \text{midpoint;}\\ \left(n_{0},r_{0}\right)=\left(n_{b},I_{0}r_{\alpha }\right), & \text{midline;}\\ \left(n,d\right)=\left(n_{s},n_{s}^{\prime }r_{\alpha }\right), & \text{midplane;} \end{array}\right. \end{array}$$ (17)

where *n*_*b*_ and *n*_*s*_ are the eigenvectors corresponding to the biggest and smallest eigenvalues of *X*_*α*_ respectively. Transformations for type 2 assessment are

$$\begin{array}{c} R=U\text{diag}\left(1,\;1,\;\text{det}\;UV^{\prime }\right)V^{\prime },\;a=\text{tr}\left(R^{\prime }A\right)/\text{tr}\left(Q^{\prime }Q\right),\;t=-aRq+t_{0}, \end{array}$$ (18)

with

$$\begin{array}{c} \left(A,t_{0}\right)=\{\begin{array}{cc} (-P^{\prime }Q,\;-p+2r_{0}), & \text{for}\;\text{point}\;\text{asymmetry;}\\ (F^{\prime }Q,\;f), & \text{for}\;\text{line}\;\text{asymmetry;}\\ (G^{\prime }Q,\;g), & \text{for}\;\text{plane}\;\text{asymmetry,} \end{array} \end{array}$$ (19)

and (*U*, *V*) coming from the singular value decomposition *A* = *UΛV*′. The general specification in (18) and (19) remains valid for special (*a*, *R*, *t*), such as (*a*, *I*, 0), (1, *R*, 0) or (1, *I*, *t*), etc. This is because *a*, *R* and *t* are considered respectively as independent scalar, matrix and vector in the minimisation.

### Assessments of asymmetry

Asymmetry assessments at a collective level are of interest in biological evolutionary studies, while at an individual level, they are useful in medical and clinical applications.

#### At individual level

The average and root-mean-square deviation (RMSD) of asymmetry indices {*e*_*i*_} from an object are

$$\begin{array}{c} e=\frac{1}{m}\sum_{i=1}^{m}e_{i}\;,\;e_{r}={\left(\frac{1}{m}\sum_{i=1}^{m}{\left\|e_{i}\right\|}^{2}\right)}^{1/2}\;. \end{array}$$ (20)

Pair (*p*_*i*_, *q*_*i*_) of an object is asymmetric if its asymmetry index *e*_*i*_ ≠ 0, and the object itself is so if at least one asymmetry index from {*e*_*i*_} is nonzero, namely *e*_*r*_ ≠ 0. In practical applications, *e*_*r*_ should be greater than a threshold which takes into account deviations from perfect symmetry caused by observational errors of asymmetry indices. If *e*_*r*_ exceeds the threshold, ‖*e*‖ indicates the extent to which the two sides of the object are unbalanced.

#### At collective level

For object *j* in a population, the set of its point pairs is denoted by {(*p*_*ij*_, *q*_*ij*_)}, and, if any, {*r*_*ij*_} the set of points shared by its both sides. The associated asymmetry index *e*_*ij*_ is calculated from (2) with (*p*_*ij*_, *q*_*ij*_) replacing (*p*_*i*_, *q*_*i*_). Accordingly, the previously defined terms *r*_*i*_, *r*_0_, (*n*_0_, *r*_0_) and (*n*, *d*) now become *r*_*ij*_, *r*_0*j*_, (*n*_0*j*_, *r*_0*j*_), and (*n*_*j*_, *d*_*j*_) respectively. If symmetry by transformation is considered, *q*_*ij*_ should be replaced by *a*_*j*_ *R*_*j*_ *q*_*ij*_ + *t*_*j*_ for all *i* and each *j*.

Point pairs {(*p*_*i*_, *q*_*i*_)} can be considered random with {(*p*_*ij*_, *q*_*ij*_)} being their samples drawn from a population. Similarly, their asymmetry indices {*e*_*i*_} defined in (2) have samples {*e*_*ij*_}. Moreover, object asymmetry index *e* defined in (20) has the averages of {*e*_*ij*_} over *i* as its sample. Asymmetries of point pairs and objects in a population are defined below, followed by their assessments.

*Asymmetry of point pairs:* Pair (*p*_*i*_, *q*_*i*_) in a population is said to be asymmetric if its asymmetry index *e*_*i*_ is statistically nonzero, namely *Pr*(‖*e*_*i*_‖ = 0) = 0, and the asymmetry is

a) fluctuating if $e_{i}∼N\left({\bar{e}}_{i},\Sigma_{i}\right)$ with ${\bar{e}}_{i}=0$;
b) directional if $e_{i}∼N\left({\bar{e}}_{i},\Sigma_{i}\right)$ with ${\bar{e}}_{i}\neq 0$;
c) antisymmetric if $e_{i}∼\gamma N\left({\bar{e}}_{ai},\Sigma_{ai}\right)+\left(1-\gamma \right)N\left({\bar{e}}_{bi},\Sigma_{bi}\right)$ with ${\bar{e}}_{ai}\neq {\bar{e}}_{bi}$ and a mixing index 0 < *γ* < 1;

where ${\bar{e}}_{i}$, ${\bar{e}}_{ia}$ and ${\bar{e}}_{bi}$ are the vector-valued means, Σ_*i*_, Σ_*ai*_ and Σ_*bi*_ the variance matrices.

*Asymmetry of objects:* Objects in a population are said to be asymmetric if object asymmetry index *e* is statistically nonzero, namely *Pr*(‖*e*‖ = 0) = 0, and the asymmetry is

a) fluctuating if $e∼N(\bar{e},\Sigma )$ with $\bar{e}=0$;
b) directional if $e∼N(\bar{e},\Sigma )$ with $\bar{e}\neq 0$;
c) antisymmetric if $e∼\gamma N\left({\bar{e}}_{a},\Sigma_{a}\right)+\left(1-\gamma \right)N\left({\bar{e}}_{b},\Sigma_{b}\right)$ with ${\bar{e}}_{a}\neq {\bar{e}}_{b}$ and a mixing index 0 < *γ* < 1;

where $\bar{e}$, ${\bar{e}}_{a}$ and ${\bar{e}}_{b}$ are the vector-valued means, Σ, Σ_*a*_ and Σ_*b*_ the variance matrices.

*Assessments*: Assessing asymmetries of point pairs and objects in a population is practically carried out by assessing the same in its samples. In respect to a sample of size *l*, denote the average and RMSD of asymmetry indices {*e*_*ij*_} over *j* by

$$\begin{array}{c} e_{i\cdot }=\frac{1}{l}\sum_{j=1}^{l}e_{ij}\;,\;e_{ir}={\left(\frac{1}{l}\sum_{j=1}^{l}{\left\|e_{ij}\right\|}^{2}\right)}^{1/2}, \end{array}$$ (21)

and those over *i* by

$$\begin{array}{c} e_{\cdot j}=\frac{1}{m}\sum_{i=1}^{m}e_{ij}\;,\;e_{rj}={\left(\frac{1}{m}\sum_{i=1}^{m}{\left\|e_{ij}\right\|}^{2}\right)}^{1/2}\;. \end{array}$$ (22)

While asymmetry index *e*_*i*_ of (*p*_*i*_, *q*_*i*_) in (2) has {*e*_*ij*_} with varying *j* as its sample, indices *e* and *e*_*r*_ in (20) have {*e*_⋅*j*_} and {*e*_*rj*_} in (22) as their samples respectively.

For assessing asymmetries of point pairs, index *e*_*ir*_ ≠ 0 approximates zero probability of ‖*e*_*i*_‖ = 0 in the population, sample mean *e*_*i*_. approximates population mean ${\bar{e}}_{i}$, and the sampling distribution of {*e*_*ij*_} approximates the population distribution of *e*_*i*_.

For assessing asymmetry of objects, indices *e*_*rj*_ ≠ 0 for all *j* in a sample approximate zero probability of ‖*e*‖ = 0 in the population, sample mean *e*_⋅*j*_ approximates population mean $\bar{e}$, and the sampling distribution of {*e*_⋅*j*_} approximates the population distribution of *e*.

In practice, distributions of *e*_*i*_ and *e* can be graphically examined with 3D scatter plots of their samples {*e*_*ij*_} and {*e*_⋅*j*_}, and/or histograms for individual components of the samples. Moreover, criteria *e*_*ir*_ ≠ 0 and *e*_*rj*_ ≠ 0 should be replaced by them exceeding some thresholds counting for observational errors of asymmetry indices.

#### Internal referencing for asymmetry index alignment

Asymmetry indices {*e*_*ij*_} are dependent on objects’ orientations. The dependence introduces artefacts to the sampling distribution of {*e*_*ij*_} since unlikely these objects have been aligned with each other when measurements of them were being taken. Removal of the artefacts can be achieved by referring sample indices {*e*_*ij*_} to an arbitrary internal frame uniformly specified for every object. This amounts to using $\left\{{\bar{R}}_{j}^{\prime }e_{ij}\right\}$ instead of {*e*_*ij*_} in asymmetry assessments, where ${\bar{R}}_{j}$ is a rotation matrix determined by points of object *j* in the same way for all objects.

A choice is ${\bar{R}}_{j}=\left[\begin{array}{ccc} r_{j}^{x} & r_{j}^{y} & r_{j}^{z} \end{array}\right]$ with its direction vectors

$$\begin{array}{c} r_{j}^{x}=\text{sgn}({\left(p_{\cdot j}-q_{\cdot j}\right)}^{\prime }n_{j})n_{j}\;,\;r_{j}^{y}=\frac{(I-r_{j}^{x}{\left(r_{j}^{x}\right)}^{\prime }){\hat{r}}_{j}}{\|(I-r_{j}^{x}{\left(r_{j}^{x}\right)}^{\prime }){\hat{r}}_{j}\|}\;,\;r_{j}^{z}=r_{j}^{x}\times r_{j}^{y} \end{array}$$ (23)

with $p_{\cdot j}=\frac{1}{m}\sum_{i=1}^{m}p_{ij}$ and $q_{\cdot j}=\frac{1}{m}\sum_{i=1}^{m}q_{ij}$. In (23), *n*_*j*_ for object *j* is the normal to the midplane determined in the same way as *n* in (17), the sign function makes $r_{j}^{x}$ point to the side of {*p*_*ij*_}, and ${\hat{r}}_{j}$ is arbitrary as long as it is not parallel to $r_{j}^{x}$, namely $|{\hat{r}}_{j}^{\prime }r_{j}^{x}\left|\neq \right\|{\hat{r}}_{j}\left\|\;\right\|r_{j}^{x}\|$. For example, as inspired by *r*_*α*_ in (6),

$$\begin{array}{c} {\hat{r}}_{j}=\frac{\left(1-\gamma \right)\sum_{i=1}^{k_{t}}r_{ij}+2\gamma \sum_{i=1}^{m_{t}}\left(p_{ij}+q_{ij}\right)}{\left(1-\gamma \right)k_{t}+4\gamma m_{t}} \end{array}$$ (24)

could be such a choice, where scalar *γ* and integers *k*_*t*_ and *m*_*t*_ are fixed for all objects with 0 ≤ *γ* ≤ 1, 0 ≤ *k*_*t*_ ≤ *k* and 0 ≤ *m*_*t*_ ≤ *m*.

Removal of the artefacts caused by external referencing from asymmetry indices can also be achieved by aligning the objects to each other through pre-processing the coordinate data, for instance, using the Procrustes superimposition. Clearly, in (21) and (22), RMSDs {*e*_*ir*_} and {*e*_*rj*_} of asymmetry indices {*e*_*ij*_} are coordinate independent. Hence, if only asymmetry but not its nature is concerned in an application, the removal or alignment is not needed.

#### Observational errors

Uncertainties in the placement and measurement of points, and inaccuracy in numerical calculation of asymmetry indices contribute to the observational errors in asymmetry analysis. To separate effects of structural deviation *ϵ* from those of observational error *ε* on asymmetry assessments, it is useful to express the points as

$$\begin{array}{c} p_{i}={\hat{p}}_{i}+\epsilon_{p,i}+\varepsilon_{p,i},\;q_{i}={\hat{q}}_{i}+\epsilon_{q,i}+\varepsilon_{q,i},\;r_{i}={\hat{r}}_{i}+\epsilon_{r,i}+\varepsilon_{r,i}, \end{array}$$ (25)

with

$$\begin{array}{c} \begin{array}{c} {\hat{p}}_{i}=f\left(p_{i},q_{i}\right),\\ {\hat{q}}_{i}=f\left(q_{i},p_{i}\right),\\ {\hat{r}}_{i}=f\left(r_{i},r_{i}\right), \end{array}\;\;f\left(a,b\right)=\{\begin{array}{cc} \left(a-b\right)/2+r_{0}, & \text{for}\;\text{point}\;\text{symmetry;}\\ \left(a+b\right)/2+I_{0}\left(r_{0}-b\right), & \text{for}\;\text{line}\;\text{symmetry;}\\ \left(a+b\right)/2+\left(d-n^{\prime }b\right)n, & \text{for}\;\text{plane}\;\text{symmetry,} \end{array} \end{array}$$ (26)

where, for simplicity, object index *j* is dropped. As the average of itself and the mirrored copy of its correspondence, ${\hat{p}}_{i}$ and ${\hat{q}}_{i}$ are perfectly symmetric about the reference on to which *r*_*i*_, if any, has projection ${\hat{r}}_{i}$.

It is reasonable to assume observational errors as noise vectors satisfying Gaussian distribution: $\varepsilon_{p,i},\;\varepsilon_{q,i},\;\varepsilon_{r,i}∼N\left(0,\sigma_{n}^{2}I\right)$ with *σ*_*n*_ being the standard deviation for each of the three components in a noise vector. If structural deviations are set to be zero (*ϵ*_*p*,*i*_ = *ϵ*_*q*,*i*_ = *ϵ*_*r*,*i*_ = 0), simulations of the simple data model (25) and (26) can generate useful information for determination of thresholds used to distinguish between effects of structural deviations and those of observational errors on asymmetry assessments.

### Flowcharts of asymmetry assessments

**Fig 2:**
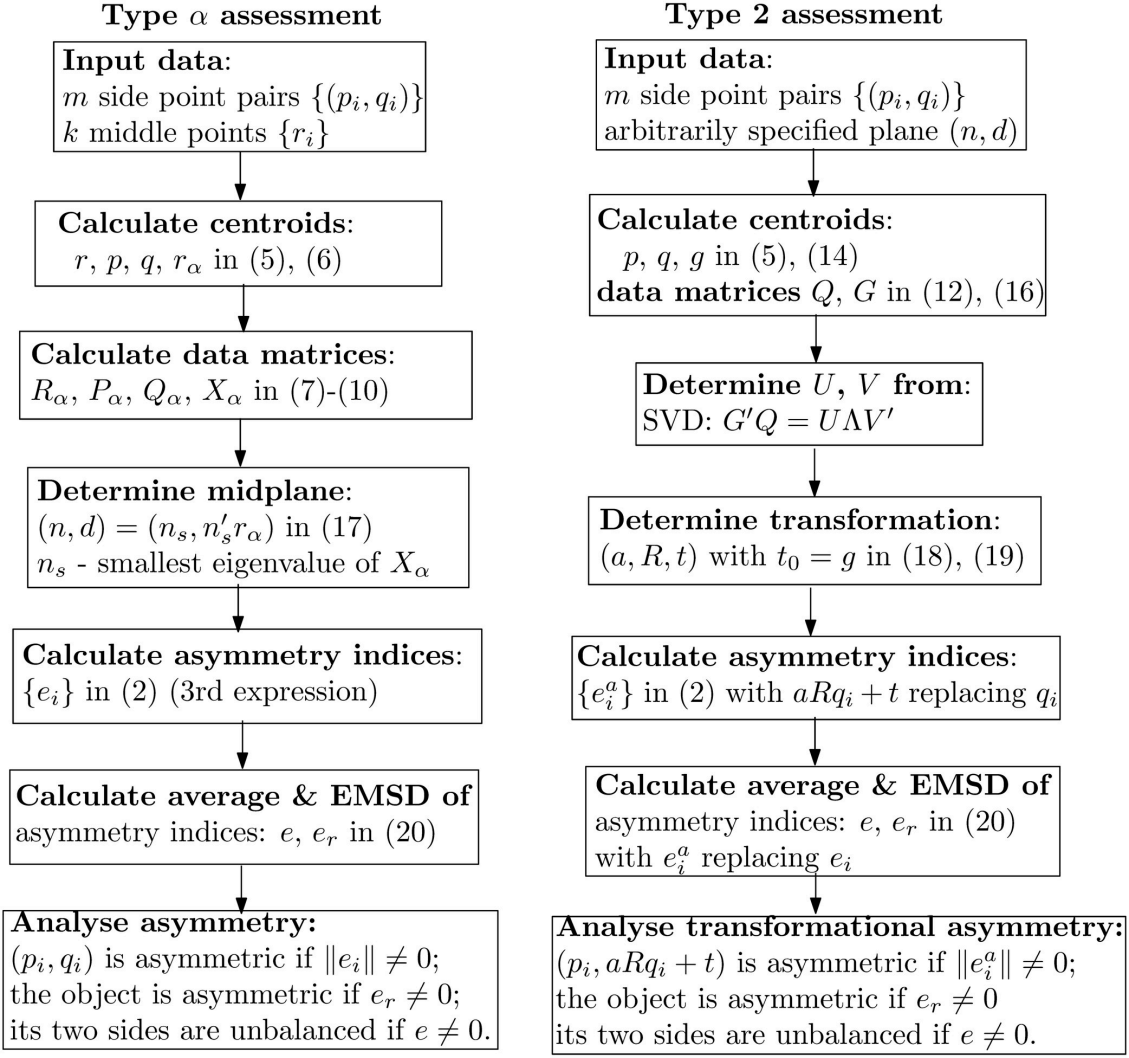
Flowcharts of plane asymmetry assessments of types *α* and 2 at individual levels.

**Fig 3:**
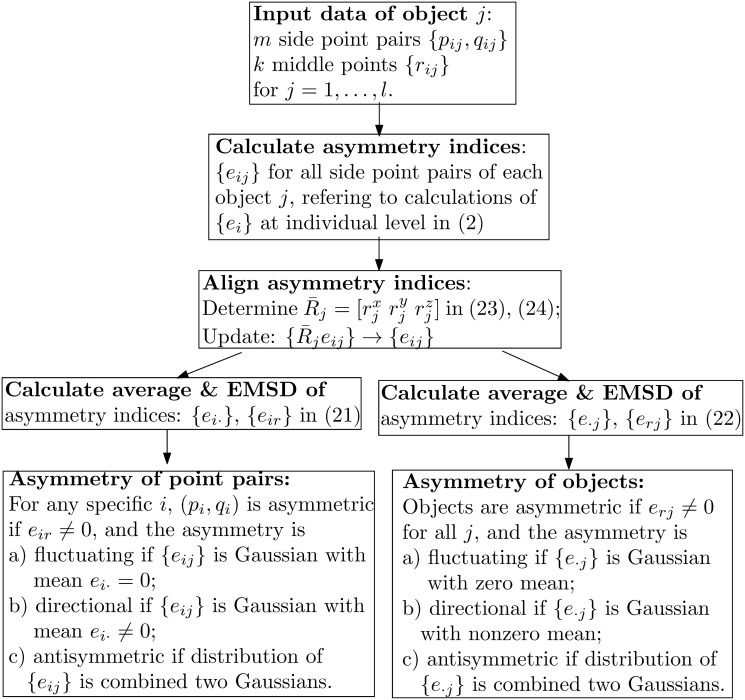
Flowchart of asymmetry assessments at collective levels.

To facilitate use of the developed scheme, three explanatory flowcharts are shown in Figs 2 and 3. The procedures in Fig 2 are for plane asymmetries at individual levels. If point or line asymmetry is considered, according to (2), (17) to (19), few straightforward adjustments are needed in the fourth block of type *α* assessment procedure, and in the first and fifth blocks of type 2 assessment procedure.

### Two testing datasets

#### Human skull dataset

**Fig 4:**
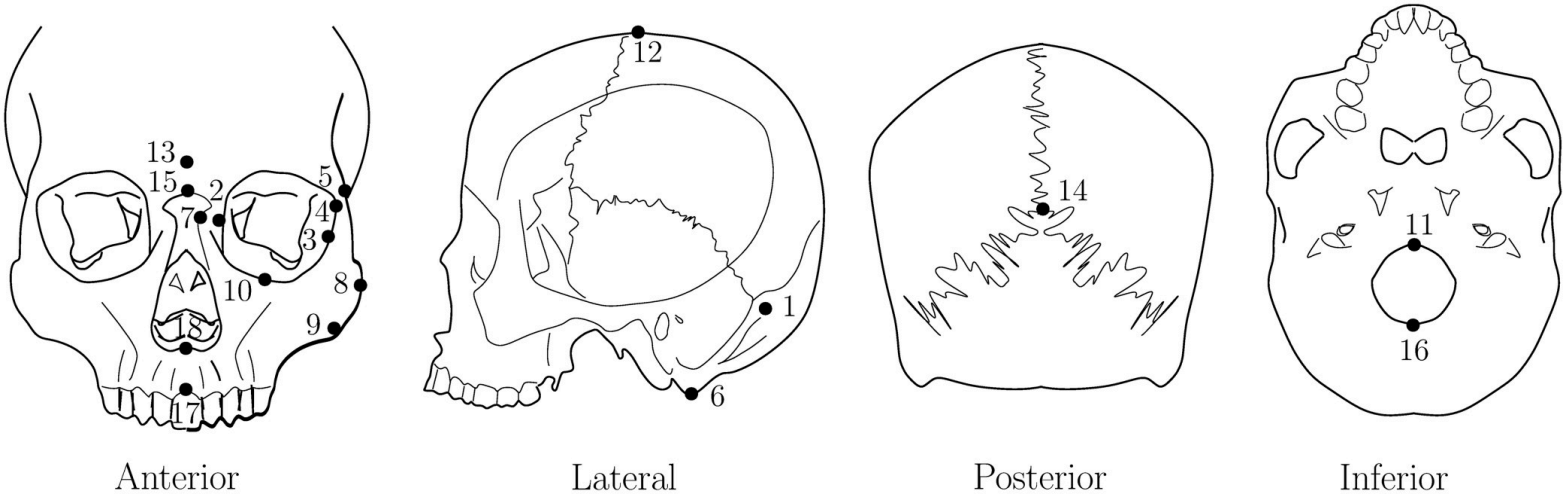
Landmarks indicated in different views of a skull.

A subset consisting of 359 skulls was selected from the collection of 889 human skulls in [21]. It includes skulls of African Americans (69 females and 71 males), and European Americans (100 females and 119 males). The only criterion for inclusion of a particular skull in this current study was the completeness of 10 pairs of side landmarks and 8 middle landmarks, as summarised in Table 1 and indicated in Fig 4. There was however one exception, namely removal of sample ID876 (European American female) due to an obvious measurement error of its left asterion. 3D landmark coordinates of the skulls in the subset obtained from the collection in [21] are used in the asymmetry assessments. To determine midplanes, the eight middle landmarks are used in type 0 assessments, while the ten side landmark pairs are used in type 1 assessments, and all of them are used in type *α* assessments. Once a midplane has been determined for asymmetry referencing, only the ten side landmark pairs need consideration and hence are used in the subsequent analysis of skull asymmetries.

**Table 1:** 2 × 10 side landmarks and 8 middle landmarks.

no	landmark (left and right)	no	landmark (middle)
1	asterion	11	basion
2	dacryon	12	bregma
3	ectroconchion	13	glabella
4	frontomalare anterior	14	lambda
5	frontomalare temporale	15	nasion
6	mastoidale	16	opisthion
7	nasomaxillary suture pinch	17	prosthion-howell
8	zygion	18	supspinale
9	zygomaxillare		
10	zygoorbitale		

#### Simulated data

This dataset contains simulated skulls whose landmarks are obtained by adding normally distributed independent random values of zero mean and a given standard deviation to each of the three landmark coordinates of the perfectly symmetric skulls. The number of the simulated skulls is the same as that of real skulls. The identical landmarks of the real skulls have been used to produce the perturbed symmetric skulls in the simulated data. A perfectly symmetric skull is represented by the landmarks described in (26). A standard deviation of 1mm for landmark displacement errors is chosen to count mainly for inaccuracy of several typical measurement methods, ranging from physical measurements by a digital calliper [22] or MicroScribe [23], to digital measurements by cone beam computed tomography [24]. By the three-sigma rule of thumb, this standard deviation corresponds to a maximal error of 3mm in landmark placement and measurement. Asymmetry indices calculated from the simulated dataset will be used as baselines for comparison with those generated from real skull dataset in asymmetry assessments.

## Results

The theoretical development of asymmetry assessments is applied to the skull datasets as a case study. In view of the nature of skull asymmetry, only plane asymmetry is examined. In type *α* assessment, *α* = 0.5 is used, and in type 2 assessment, the midplane is the one determined in type 1 assessment. For alignment of asymmetry indices, parameters *γ* = 1/2, *k*_*t*_ = *k*/2 and *m*_*t*_ = *m*/2 are used in (24). The abbreviations for categorisation are AA: African American, EA: European American, F: Females, M: Males.

In some plots, where asymmetries of 359 individual skulls are concerned, the horizontal axes represent skull indices up to that total number. In other plots, when comparisons among the four skull categories are considered, the skull indices shown for the horizontal axes are only up to 119 which is for EA male skulls, the largest number of the skulls in the four categories.

### Noise effects on symmetry

With respect to different assessment types, landmark-pair asymmetry of perturbed symmetric skulls caused by observational errors are produced. As expected, at the population level, asymmetry of every landmark pair of perturbed symmetric skulls is fluctuating. S1 Fig in S2 Appendix indicates the landmark-pair asymmetry of perturbed symmetric skulls caused by observational errors. As an example of type 1 assessment, scattered points and histograms of asymmetry index of the 6th landmark pair are shown in S2 Fig in S2 Appendix.

With respect to different assessment types, the first sub-figure in S3 Fig in S2 Appendix indicates indistinguishable variations in the RMSDs of asymmetry indices for the perturbed symmetric skulls. Scatter plots in S3 Fig in S2 Appendix show distribution ranges of skull asymmetry indices {*e*_⋅*j*_} with respect to different assessment types. These responses from the perturbed symmetric skulls provide comparing baselines for asymmetry assessments of the real skulls. As will be seen, in general, the baseline asymmetry indices are minor compared with large magnitudes of asymmetry indices from the real skulls.

### Landmark-pair asymmetry

**Fig 5:**
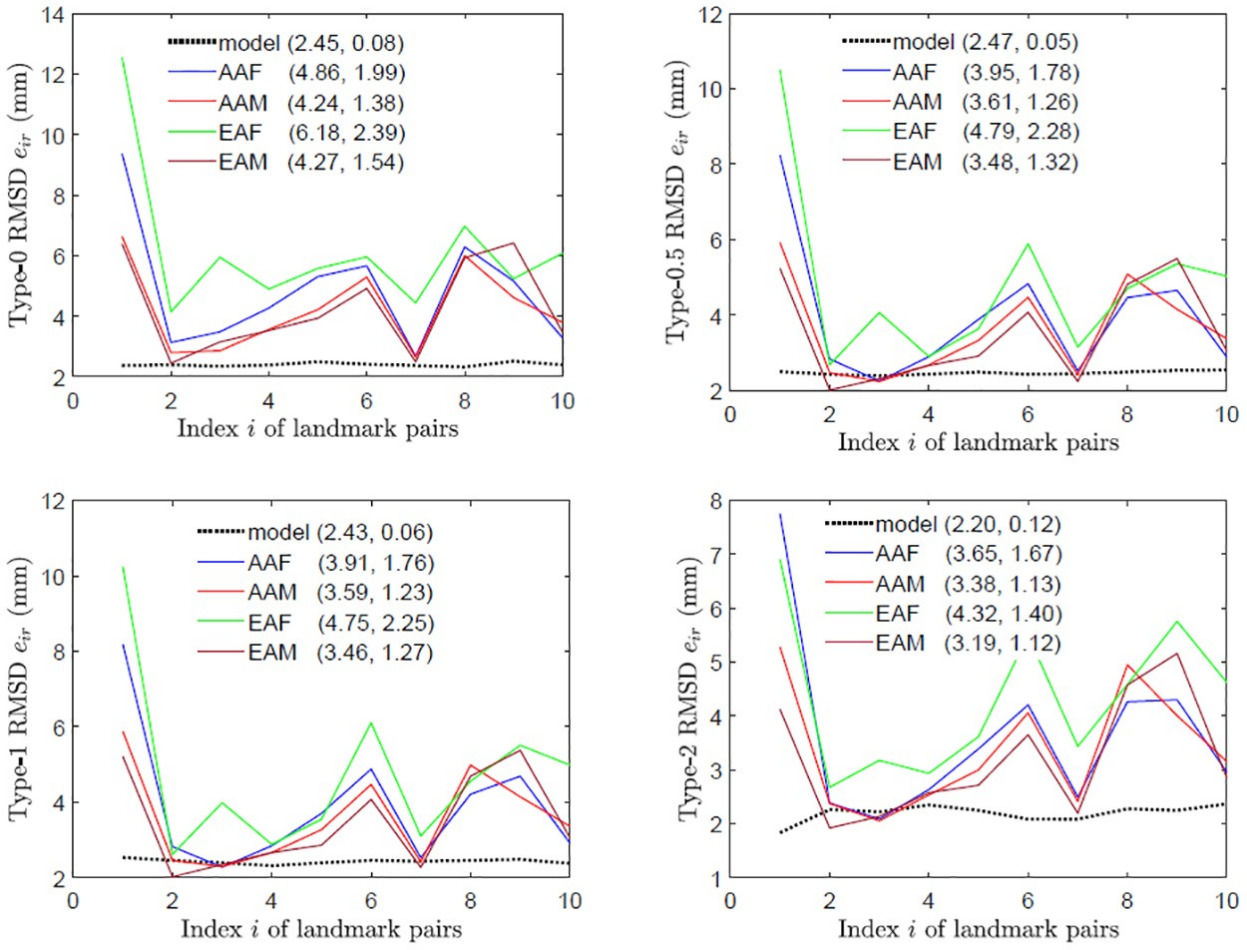
Asymmetry of landmark pairs. The plots are for the perturbed symmetric skulls (model) and original skulls with respect to different assessment types (0, 0.5, 1 and 2) and in different categories (AAF, AAM, EAF and EAM), where in brackets are the means and standard deviations of the RMSDs for asymmetry indices.

The RMSDs in Fig 5 suggest overall asymmetries in most of the 10 landmark pairs, induced by bioecological factors in the studied human skulls. In particular, landmark pairs of female skulls appear more asymmetric than those of male skulls. Also, as shown in S4 Fig in S2 Appendix, asymmetry indices of landmark pairs of female skulls have more variations than those of male skulls do.

**Fig 6:**
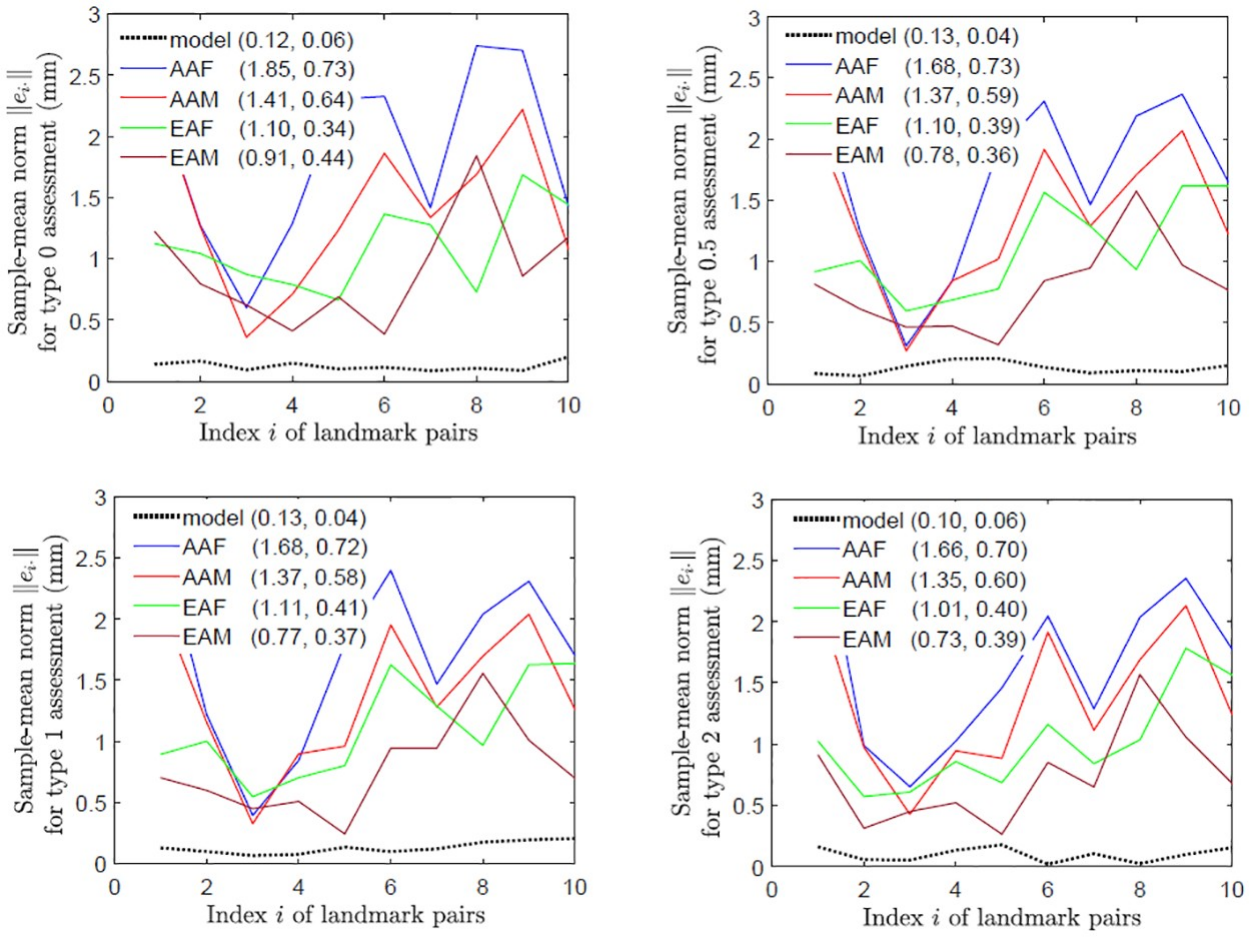
Non-fluctuating asymmetry of landmark pairs. The plots are for the perturbed symmetric skulls (model) and original skulls with respect to different assessment types (0, 0.5, 1 and 2) and in different categories (AAF, AAM, EAF and EAM), where in brackets are the means and standard deviations of the sample-mean norms of asymmetry indices.

**Fig 7:**
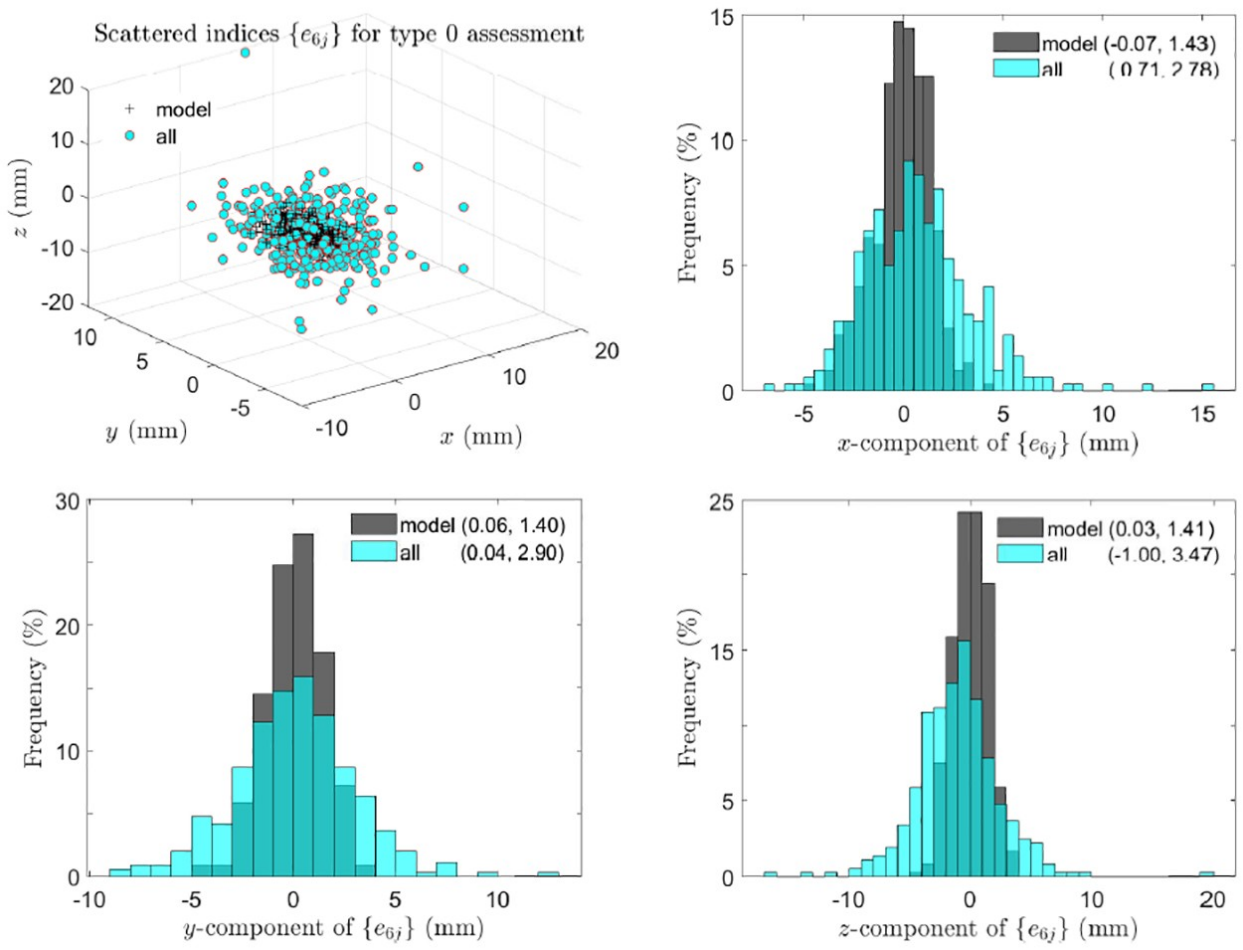
Type 0 assessment: Directional asymmetry of landmark pairs. Scattered points and component histograms of asymmetry indices {*e*_6*j*_} in type 0 assessment from the 359 perturbed symmetric skull and all real skulls are shown, where in brackets are the means and standard deviations of components of {*e*_6*j*_}.

Fig 6 indicates that asymmetry of landmark pairs is not fluctuating. As an example, for type 0 assessment, scattered points and component histograms of asymmetry indices of the 6th landmark pair are shown in Fig 7, where the components of the asymmetry indices appear to follow slightly skewed normal distributions with clearly non-zero means of *x* and *z* components. Along with similar plots of scattered points and histograms for types 1 and 2 assessments shown in S5 and S6 Figs in S2 Appendix, these plots indicate that the 6th landmark pair is directionally asymmetric.

### Skull asymmetry

**Fig 8:**
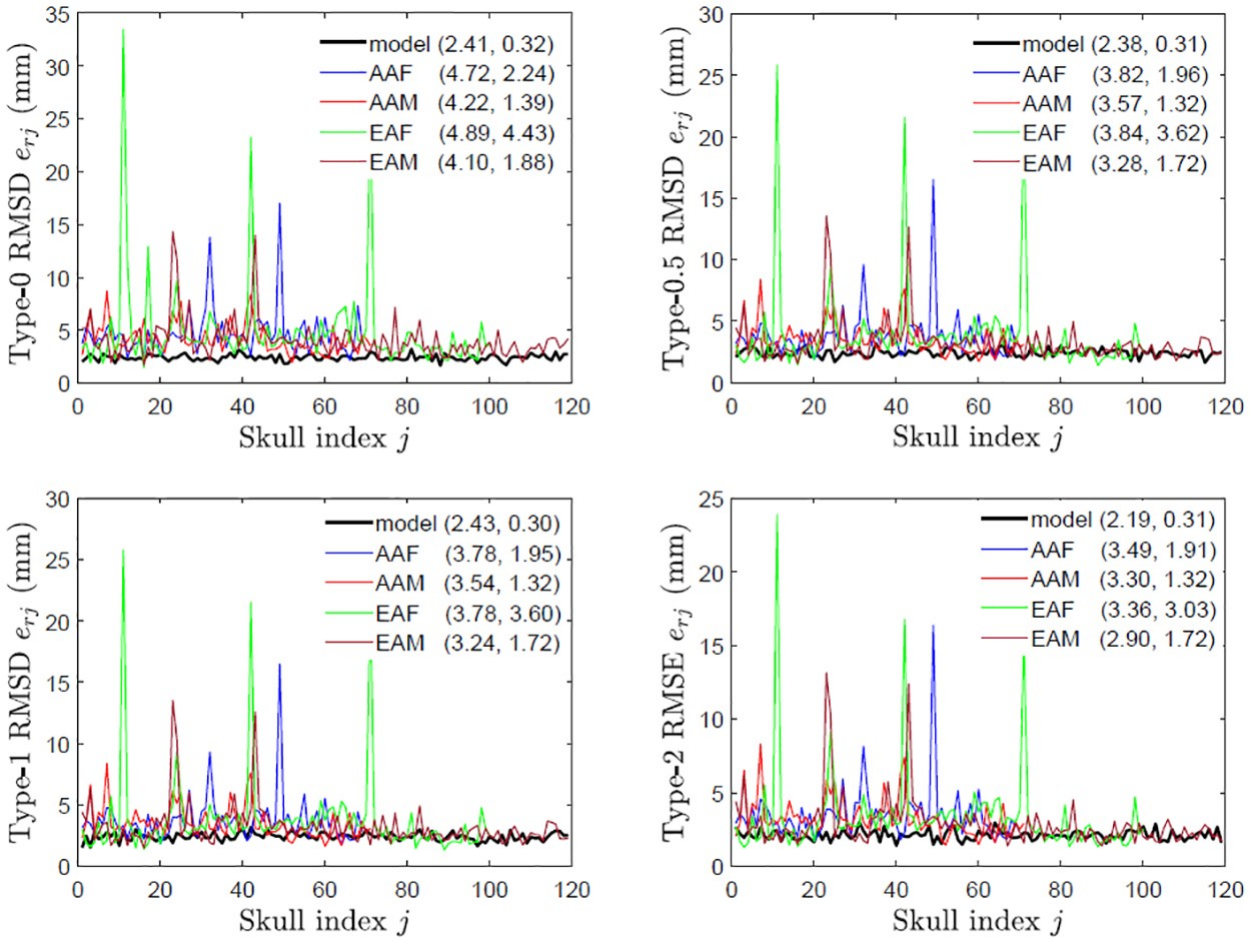
Skull asymmetry in different categories. RMSDs of asymmetry indices of the first 119 perturbed symmetric skulls (model) and original skulls with respect to different assessment types (0, 0.5, 1 and 2) and in different categories (AAF, AAM, EAF and EAM) are shown, where in brackets are the means and standard deviations of the RMSDs.

**Fig 9:**
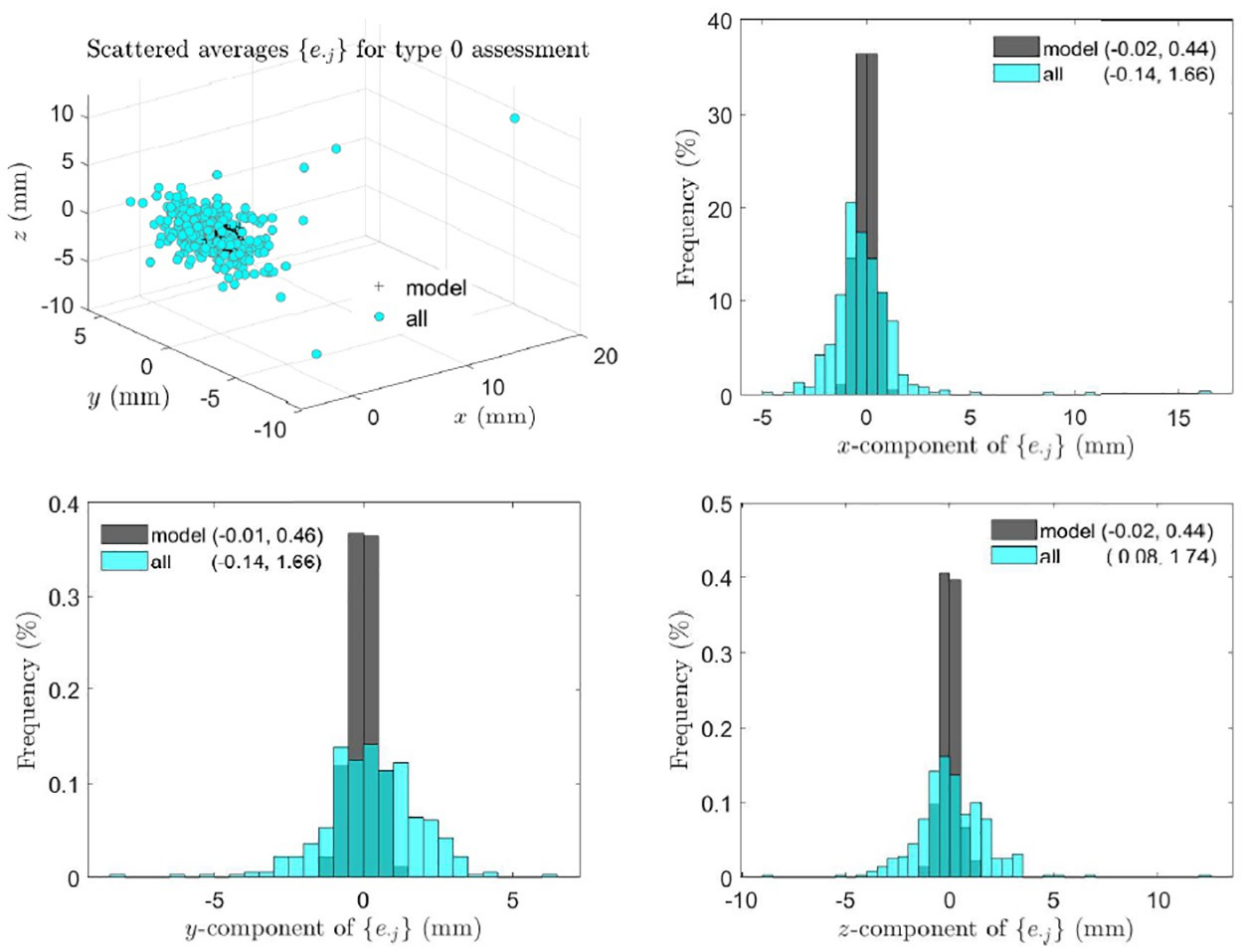
Type 0 assessment: Directional asymmetry of skulls. Scattered points and histograms of asymmetry indices of the 359 perturbed symmetric skull and all real skulls are shown, where in brackets are the the means and the standard deviations of components of {*e*_⋅*j*_}.

**Fig 10:**
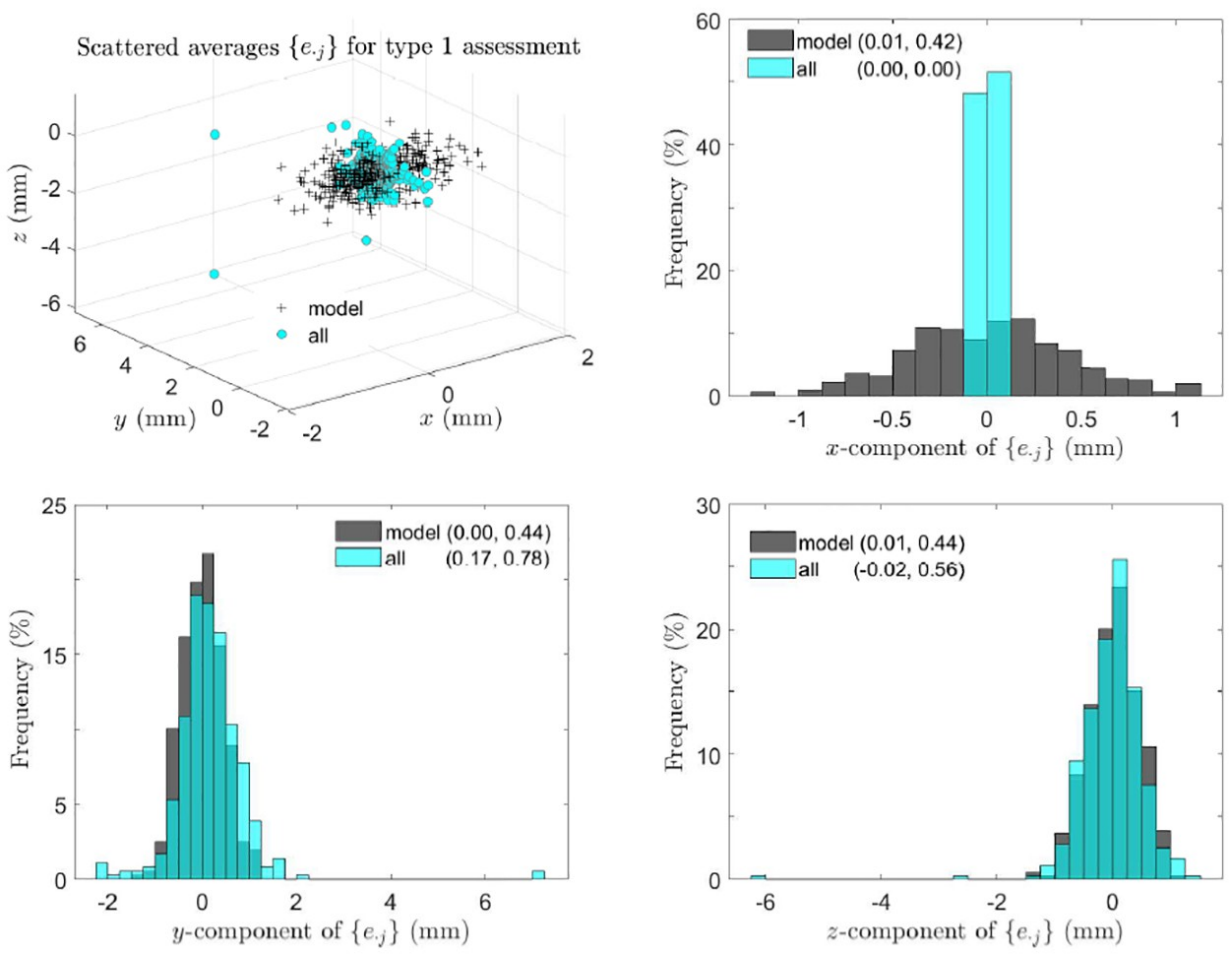
Type 1 assessment: Directional asymmetry of skulls. Scattered points and histograms of asymmetry indices of the 359 perturbed symmetric skull and all real skulls are shown, where in brackets are the the means and standard deviations of components of {*e*_⋅*j*_}.

**Fig 11:**
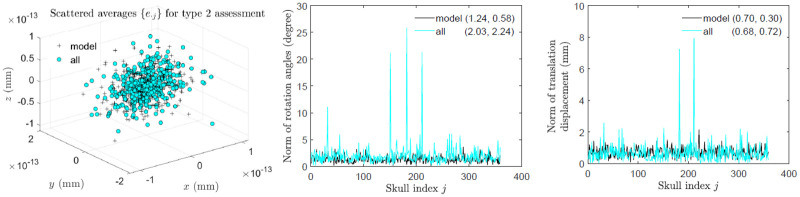
Type 2 assessment: Fluctuating asymmetry of skulls. Scattered points of averaged asymmetry indices and norms of rotation angles and translation displacements of the 359 perturbed symmetric skull and all real skulls are shown, where in brackets are the means and standard deviations of the norms.

Fig 8 shows that the majority of the skulls are asymmetric and on average female skulls are more asymmetric than male skulls. In type 0 assessment, Fig 9 shows that the skull asymmetry is not fluctuating, but directional due to clear deviations of the mean of {*e*_⋅*j*_} from that of perturbed symmetric skulls. In type 1 assessment, Fig 10 shows that the asymmetry remains directional since in *y*-direction the mean of {*e*_⋅*j*_} is much greater than that of perturbed symmetric skulls although the *x* and *z* components of the mean are close to those of perturbed symmetric skulls. Interestingly, in type 2 assessment shown in Fig 11, the skull asymmetry becomes completely fluctuating since the scale of the scatter plot (first plot) remains at 10^−13^ mm as in the third scatter plot of S3 Fig in S2 Appendix, and the amounts of the associated rotations and translations are very small in general, with the means at 2° and 0.7 mm respectively. There are however 3 or 4 outliers (about 1% of the total skulls) requiring much larger rotational and/or translational adjustments. The angular norm in Fig 11 is calculated from the three rotation angles as a vector associated with each rotation matrix. These angles are converted from the quaternions which are first calculated from each rotation matrix.

### Procrustes analysis

As detailed in the Discussion section, few straightforward adjustments of the specification of type 2 assessment in (18) and (19) give rise to explicit solutions needed in the Procrustes analysis. Use of these explicit and direct transformations easily avoids undesirable rotations and reflections in the orthogonal Procrustes transformation and superimposition used for object reflection and alignment respectively.

For object symmetry assessment, midplane (*n*_*j*_, *d*_*j*_) for referencing object *j* is the one used for type 0 assessment, and the superimposition is applied to $\left\{{\bar{p}}_{ij}\right\}$ and {*p*_*ij*_}, where ${\bar{p}}_{ij}=q_{ij}+2\left(d_{j}-n_{j}^{\prime }q_{ij}\right)n_{j}$ represents the reflection of *q*_*ij*_ through the *j*th midplane. For matching symmetry assessment, reflection *R*_*j*_ and translation *t*_*j*_ are determined for object *j* first and the superimposition is then applied to {*R*_*j*_*q*_*ij*_ + *t*_*j*_} and {*p*_*ij*_}. Accordingly, after the reflections and superimpositions, assessment indices $e_{ij}^{o}={\bar{p}}_{ij}-p_{ij}$ and $e_{ij}^{m}=R_{j}q_{ij}+t_{j}-p_{ij}$ are used for object and matching asymmetries respectively. In the superimposition, all objects are aligned to the first skull, namely the ordinary Procrustes analysis is used.

Although the procedure described above may not be exactly the same as those adopted by other investigators in applications of the Procrustes method to asymmetry assessments, it effectively captures the essence of the method and links it to the scheme developed in the current study.

**Fig 12:**
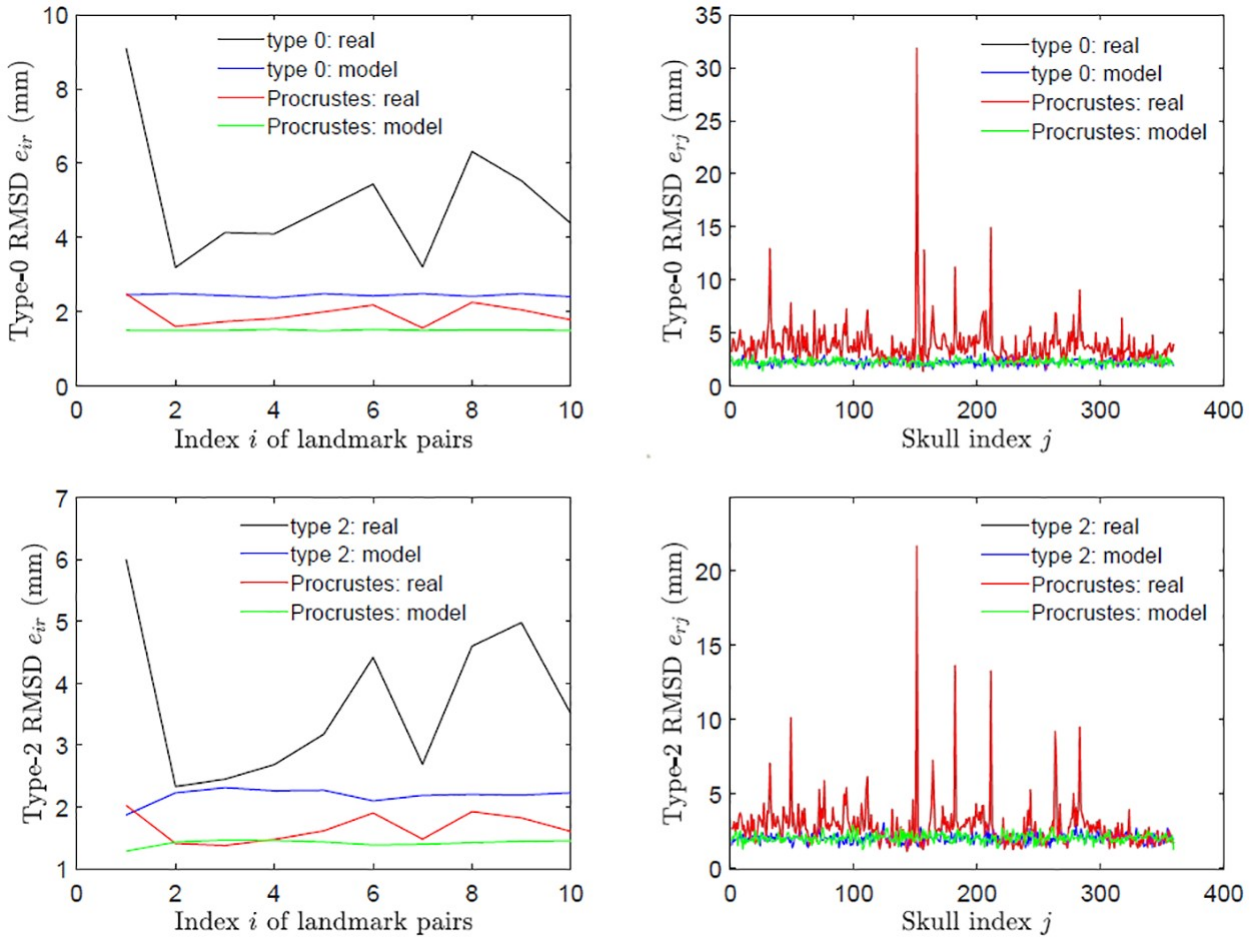
A comparison with the Procrustes analysis. The plots are for the perturbed symmetric skulls (model) and original skulls (real) with respect to type 0 assessment (top) and type 2 assessment (bottom) in this work, and object symmetry (top) and matching symmetry (bottom) of Procrustes method; RMSDs of asymmetry indices of landmark pairs (left), and objects (right).

Fig 12 shows a brief comparison between type 0 and 2 assessments and the Procrustes analysis of asymmetries of landmark pairs and objects for the perturbed symmetric skulls and original skulls. Two observations and some comments are made below:

a) In asymmetry assessment of landmark pairs, both methods work well for producing the RMSDs of asymmetry indices for real skulls distinguishable from those for perturbed symmetric skulls. The difference between real and model skull RMSDs is greater in the current method than in the Procrustes method. This also true after scaling (not shown).
b) In asymmetry assessment of skulls, both methods produced the identical RMSD of asymmetry indices for real skulls, and comparable ones for perturbed symmetric skulls.

The adjustments to the Procrustes analysis as explained above avoid making it unfairly disadvantaged in such a brief comparison. This leads to equivalence of the two methods when used for assessing asymmetries of objects, as confirmed by observation b). Although observation a) indicates validity of both methods for assessing asymmetries of point pairs of these objects, it does suggest an advantage of the current method over the other. The reason behind this lies in different alignments of asymmetry indices by the two methods. Since object alignment plays no role in asymmetry assessment of individual skulls, it is logic to have observation b). Recall that in Figs 9–11, examination of distributions of skull asymmetry indices {*e*_⋅*j*_} requires removal of the artefacts induced by unknown orientations of the objects, alignment of these indices for all skulls in the sample has been used as well according to (23) and (24).

As a final remark, the Procrustes method could be improved by using the generalised Procrustes superimposition which is an iterative procedure. A detailed study of that is beyond the scope of the current work.

## Discussion

### General consideration

In an analytic geometry setting, this study has rigorously defined several asymmetry assessments and derived explicit solutions to them. Recast in the framework of analytic geometry, notions of fluctuating asymmetry, directional asymmetry and antisymmetry have also been refined. The main computations used for the asymmetry assessments are calculations of the eigenvalue/eigenvector and singular value decomposition of data matrices. Most popular software packages for numerical calculations can be used to complete these computations. The methods have been generally developed for 3D objects, but are applicable with minor adjustments to 2D objects, where point and/or line asymmetry is concerned. An extension of the treatments to *n*D objects for *n* > 3 is also possible, which is of theoretical interest at least.

The illustration of the assessments in a study of human skull bilateral asymmetry shows the efficiency of the methods. Effects of uncertainties in the landmark placement and measurement on asymmetry assessment have been examined, which helps differentiate the effects of observational errors and bioecological factors. The current study assumes that the correspondences between points in the two halves of an object are known. If this is not the case, permutations of points in one half of the object need to be combined with asymmetry assessments. However, the developed treatments do not apply to an object with different numbers of landmarks on its two sides and in particular when no clear correspondence between any subsets of the points on its two has been established.

### Assessment types

Different assessment types evaluate bilateral asymmetry of an object from different perspectives. Type 0 assessment for evaluation of deviations from point, line or plane symmetry is useful when there are middle points centred around a midpoint, midline or midplane. Type 1 assessment applies to the situations where no middle points are recorded, or they are not well centred and better interpreted as being scattered around a midpoint, midline or midplane. As a trade-off between types 0 and 1, type *α* could be more convenient and useful for asymmetry assessment in practical applications since types 0 and 1 are its special cases. Type 2 assessments are normally used for examining asymmetries of objects with two disjoint sides.

If an object is examined sequentially by all three assessment types 0, 1 and 2, asymmetry indices will be largest for type 0 assessments and smallest for type 2 assessments, and the asymmetry index associated with type *α* assessment is normally between those of types 0 and 1. While the solution of fitting a line or plane to a set of points used in type 0 assessment is known and particularly implied in a general solution to linear manifold fitting [25], a simple direct derivation of type *α* and 2 assessments for point, line and plane asymmetries can be found in S1 Appendix. With respect to the categories of *object symmetry* and *matching symmetry* [14, 20], type *α* belongs to the former, while type 2 to the latter.

### Relationship with Procrustes method

In [14], notions of the object symmetry and matching symmetry in terms of reflection and permutational transformations on a data matrix were formulated, and asymmetry assessments were based on statistical and geometrical analyses of the Procrustes shape manifold. The Procrustes method is normally used to assess object and matching symmetries [20]. Originally developed for studying similarity of objects, the well-known Procrustes method, see e.g. [26, 27], allows rotation or reflection along with scaling and translation to be applied to points on one side to be compared with those on the other. Conceptually symmetry is secondary to similarity, which makes a general method developed for one not best suit the other.

In the current study, through direct formulations in 3D analytic geometry, the object symmetry is assessed by type *α* with 0 ≤ *α* ≤ 1, and the matching symmetry by type 2 assessments of deviations from point, line or plane symmetry. An advantage of the current treatment is its direct formulation with explicit solutions. As shown in S1 Appendix, type 2 assessment is readily reducible to a minor variation of the standard orthogonal Procrustes and Wahba’s problems [28–30]. Also, as explained below, with few straightforward adjustments, the transformation specified for type 2 assessment becomes transformations that best meet the needs of asymmetry analysis using the Procrustes method.

In general, with or without translation, rotation of one of two identical halves does not make a symmetric pair, and conversely, reflection of half of a symmetric pair breaks the symmetry. Hence, in asymmetry analysis, only reflections with translations in the Procrustes transformations should be kept, which can be ensured by simply setting *a* = 1, *g*_*i*_ = *p*_*i*_ and using slightly adjusted *R* = *Udiag*(1, 1, −*detUV*′)*V*′ in the solution (*a*, *R*, *t*) of type 2 assessment given in (18) and (19). The adjustments amount to re-defining type 2 assessment as to find a reflection along with a translation of points on one object side so that the transformed points become closest to those on the other. For the purpose of asymmetry assessment, this re-definition is mathematically equivalent to the original one. Similarly, for aligning two objects, only rotations with translations in the Procrustes superimposition should be retained, which can be ensured by the transformation (*a*, *R*, *t*) of type 2 assessment if all points of one object are re-denoted by {*g*_*i*_}, and those of another by {*q*_*i*_} in (18) and (19).

### Asymmetry of human skulls

Asymmetry of mammalian skulls is well known, see, e.g. [31, 32]. Studies of asymmetry of human skulls have initially used length and angle measurements and lately the Procrustes analysis of landmarks, see, e.g. [12, 17, 33–35]. The current study shows that with respect to a midplane fitted to the shared landmarks (type 0 assessment) or to side landmarks for balancing the two sides (type 1 assessment), asymmetry of the studied human skulls is generally directional, which is consistent with the previous discoveries in the literature (e.g. [33, 34]). In general, in the studied population, female skulls appear clearly more asymmetric than male skulls. Nevertheless, no significant cranium directional asymmetry differences were found in [35] between the sexes in a modern Greek population. Finally, the current study shows that, after a gentle coordinate transformation (type 2 assessment) is applied to the landmarks of one side, the skull asymmetry becomes extraordinarily fluctuating, and the associated rotational angles and translational displacement are minor. This interesting feature does not seem to have been reported elsewhere.

### Other applications

Apart from analysis of rotational and translational symmetries for discovery of the nature of bilateral symmetry and exploration of scaling, radial and glide symmetries, type 2 assessment of matching symmetry is useful in other applications. For instance, forensic and archaeological investigations are often interested in matching one part of an object to another from a number of candidates. In medical and clinical applications, a large deviation from symmetry of two sides of an organ can be a diagnostic factor for certain diseases affecting that organ. Alignment of one object with another to make a symmetric pair is likely required in various surgical operations. Investigations on asymmetry of species in evolutionary biological studies at macroscopic and microscopic levels benefit research on developmental instability and its causes in a population. The number of publications on asymmetry assessments in these diverse disciplines is huge, and specific referencing is spared here. The current study has attempted to provide investigators in wide application areas of asymmetry assessments with a solid and transparent analytical base.

## Supporting information

S1 Appendix Derivation of Eqs (17) to (19) [36]. (PDF)

S2 Appendix Additional simulation results. (PDF)
