# Supplementary material for: Assessments of bilateral asymmetry with application in human skull analysis
Source: PLoS One. 2021 Oct 6;16(10):e0258146. doi: 10.1371/journal.pone.0258146 (PMC8494363; doi:10.1371/journal.pone.0258146)
Supplement: S1 Appendix — (PDF) [file pone.0258146.s001.pdf]

S1 Appendix: Derivation of Eq (17) to Eq (19)

Refer to the equations in (1) to (19) for the notations and associated specifications.

Point asymmetry

For type α, minimising J_α is to have the least squares solution of r_0 to

$$\sqrt{1-\alpha}(r_i - r_0) = 0, \quad \sqrt{\alpha}(q_j + p_j - 2r_0) = 0$$

for $i = 1, 2, \ldots, k$, and $j = 1, 2, \ldots, m$. Note that, in general, a least squares solution to these equations is different from that without weightings $\sqrt{1-\alpha}$ and $\sqrt{\alpha}$. Stacking these equations yields $Ar_0 = Bb_0$ with

$$\begin{aligned} A &= \left[\ \sqrt{1-\alpha}I, \ \cdots, \ \sqrt{1-\alpha}I, \ 2\sqrt{\alpha}I, \ \cdots, \ 2\sqrt{\alpha}I\ \right]', \\ B &= \operatorname{diag}\left(\sqrt{1-\alpha}I, \ \cdots, \ \sqrt{1-\alpha}I, \ \sqrt{\alpha}I, \ \cdots, \ \sqrt{\alpha}I\right), \\ b_0 &= \left[\ r_1', \ \cdots, \ r_k', \ p_1' + q_1', \ \cdots, \ p_m' + q_m'\ \right]'. \end{aligned}$$

Since $A^+ = A'/c$ with $c = (1-\alpha)k + 4\alpha m$, the least squares solution is $r_0 = A^+ B b_0 = r_\alpha$.

For type 2, minimising J_2 with respect to t is to have the least squares solution of t to $aRq_i + t + p_i = 2r_0$ for $i = 1, 2, \ldots, m$, which readily leads to $t = -aRq - p + 2r_0$. With this t, $J_2 = \sum_{i=1}^m \|aR(q_i - q) + (p_i - p)\|^2$, which is a minor variation of the standard orthogonal Procrustes and Wahba's problems [29, 30, 36], and verification of the solution (a, R, t) in this case is straightforward [28].

Line asymmetry

For type α, minimising J_α is to have the least squares solution of r_0 to

$$\sqrt{1-\alpha}I_0\,(r_i - r_0) = 0, \quad \sqrt{\alpha}\,(q_j + p_j - 2r_0 - 2n_0'(p_j - r_0)n_0) = 0$$

for $i = 1, 2, \ldots, k$, and $j = 1, 2, \ldots, m$. Stacking these equations yields $AI_0 r_0 = B(b_0 - 2b_1)$ with

$$b_1 = \left[\ 0, \ \cdots, \ 0, \ n_0' p_1' n_0, \ \cdots, \ n_0' p_m' n_0\ \right]',$$

and A, B and b_0 being defined in the case of point symmetry. From $I_0^+ = I_0$ and $I_0^2 = I_0$, the least squares solution is then given by $r_0 = (AI_0)^+ B(b_0 - 2b_1) = I_0 A^+ B(b_0 - 2b_1) = I_0\left(r_\alpha - \frac{4\alpha m n_0' p}{(1-\alpha)k + 4\alpha m} n_0\right) = I_0 r_\alpha$.

Denote $\tilde{r}_i = r_i - r_\alpha$, $\tilde{p}_i = p_i - r_\alpha$ and $\tilde{q}_i = q_i - r_\alpha$. To determine n_0, substituting r_0 into J_α yields

$$\begin{aligned} J_\alpha &= (1-\alpha)\sum_{i=1}^k \|\tilde{r}_i - n_0'\tilde{r}_i n_0\|^2 + \alpha\sum_{i=1}^m \|\tilde{q}_i + \tilde{p}_i - 2n_0'\tilde{p}_i n_0\|^2 \\ &= (1-\alpha)\sum_{i=1}^k (\tilde{r}_i'\tilde{r}_i - n_0'\tilde{r}_i\tilde{r}_i' n_0) + \alpha\sum_{i=1}^m ((\tilde{p}_i + \tilde{q}_i)'(\tilde{p}_i + \tilde{q}_i) - 2n_0'(\tilde{p}_i\tilde{q}_i' + \tilde{q}_i\tilde{p}_i')n_0) \\ &= \operatorname{tr}((1-\alpha)R_\alpha' R_\alpha + \alpha(P_\alpha + Q_\alpha)'(P_\alpha + Q_\alpha)) - n_0' X_\alpha n_0. \end{aligned}$$

Setting $\frac{\partial J}{\partial n_0} = 0$ with $J = J_\alpha + \lambda(1 - n_0' n_0)$ leads to $(\lambda I - X_\alpha)n_0 = 0$. This means that with n_0 being the unit eigenvector corresponding to the largest eigenvalue value λ of X_α, $n_0' X_\alpha n_0$ reaches the maximum, and hence J achieves the minimum.

For type 2, the least squares solution of t to $aRq_i + t - f_i = 0$ for $i = 1, 2, \ldots, m$, is $t = f - aRq$. With this t, $J_2 = \sum_{i=1}^{m} \|aR(q_i - q) - (f_i - f)\|^2$. As in the case of point symmetry, the solution in this case follows.

Plane asymmetry

For type α, to minimise $J = J_\alpha + \lambda(1 - n'n)$, setting $\frac{\partial J}{\partial d} = 0$ gives

$$(1-\alpha)\sum_{i=1}^{k}(n'r_i - d) + 2\alpha\sum_{i=1}^{m}\left(q_i - p_i - 2n(d - n'p)\right)' n = 0$$

which has the solution $d = n'r_\alpha$. Substituting d into J_0 and J_1 produces

$$\begin{aligned} J_\alpha &= (1-\alpha)\sum_{i=1}^{k}(n'\tilde{r}_i)^2 + \alpha\sum_{i=1}^{m}\|\tilde{q}_i - \tilde{p}_i + 2n'\tilde{p}_i n\|^2 \\ &= (1-\alpha)\sum_{i=1}^{k} n'\tilde{r}_i\tilde{r}_i' n + \alpha\sum_{i=1}^{m}\left((\tilde{q}_i - \tilde{p}_i)'(\tilde{q}_i - \tilde{p}_i) + 2n'(\tilde{p}_i\tilde{q}_i' + \tilde{q}_i\tilde{p}_i')n\right) \\ &= \mathrm{tr}\left(\alpha(P_\alpha - Q_\alpha)'(P_\alpha - Q_\alpha)\right) + n'X_\alpha n. \end{aligned}$$

In view of the verification of type α for point and line symmetries, this means that with n being the unit eigenvector corresponding to the smallest eigenvalue value λ of X_α, $n'X_\alpha n$ reaches the minimum, and so do J_α and J.

For type 2, the verification of solution (a, R, t) in this case is exactly the same as that in line symmetry, but with g_i replacing f_i in J_2.
