# Supplementary material for: Assessments of bilateral asymmetry with application in human skull analysis
Source: PLoS One. 2021 Oct 6;16(10):e0258146. doi: 10.1371/journal.pone.0258146 (PMC8494363; doi:10.1371/journal.pone.0258146)
Supplement: S2 Appendix — (PDF) [file pone.0258146.s002.pdf]

S2 Appendix: Additional simulation results

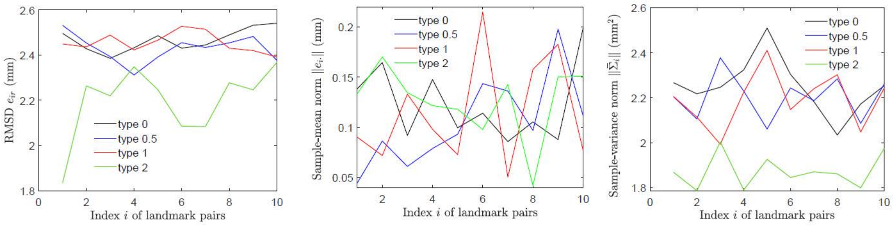

Fig S1. Asymmetry of perturbed landmark pairs. RMSDs (left) and norms of sample mean (middle) and sample variance (right) of asymmetry indices of the ten landmark pairs from the 359 perturbed symmetric skulls.

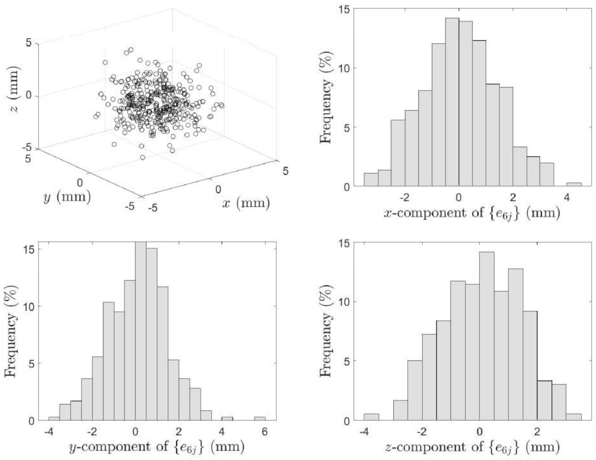

Fig S2. Fluctuating asymmetry of perturbed landmark pairs. Scattered points and histograms of asymmetry indices of type 1 assessment for the 6th landmark pair from the 359 perturbed symmetric skulls

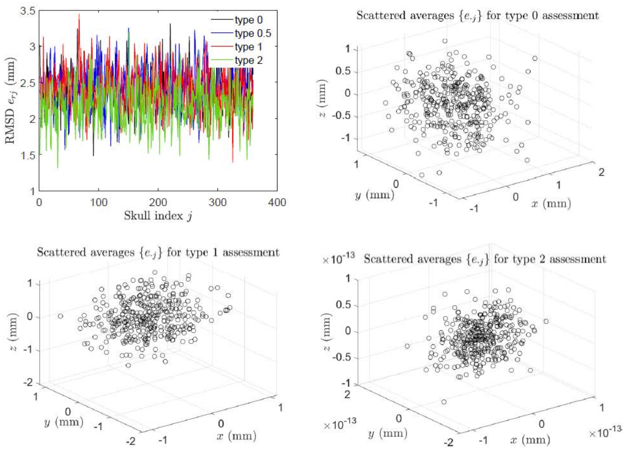

Fig S3. Sampling distribution of asymmetry indices for perturbed symmetric skulls. RMSDs and scattered plots for averages $\{e_{\cdot j}\}$ of the 359 perturbed symmetric skulls

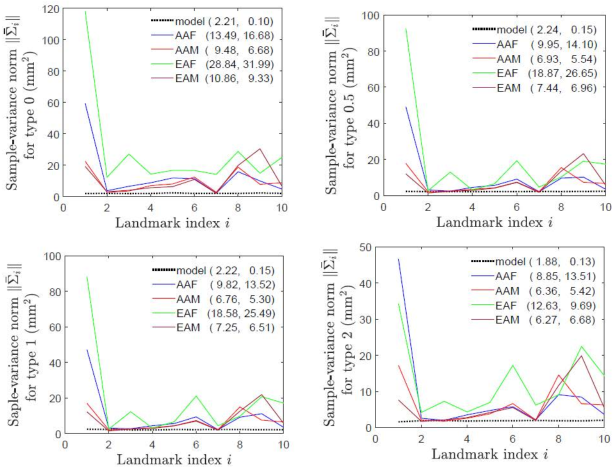

Fig S4. Sample variances of landmark-pair asymmetry indices. The plots are for the perturbed symmetric skulls (model) and original skulls with respect to different assessment types (0, 0.5, 1 and 2) and in different categories (AAF, AAM, EAF and EAM), where in brackets are the means and standard deviations of the sample-variance norms.

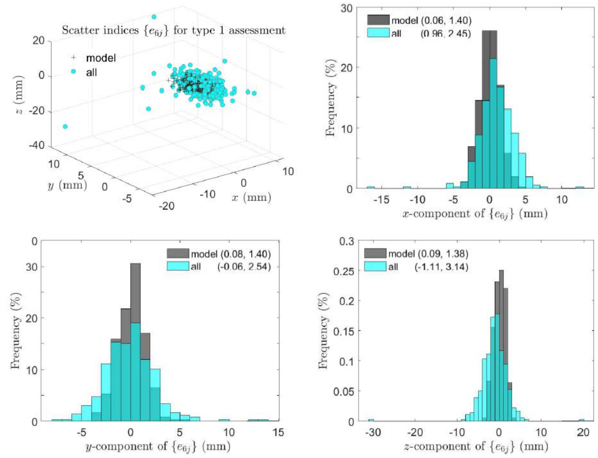

Fig S5. Type 1 assessment: Directional asymmetry of landmark pairs. Scattered points and histograms of asymmetry indices of $\{e_{6j}\}$ from the 359 perturbed symmetric skull and all real skulls are shown, where in brackets are the means and standard deviations of components of $\{e_{6j}\}$.

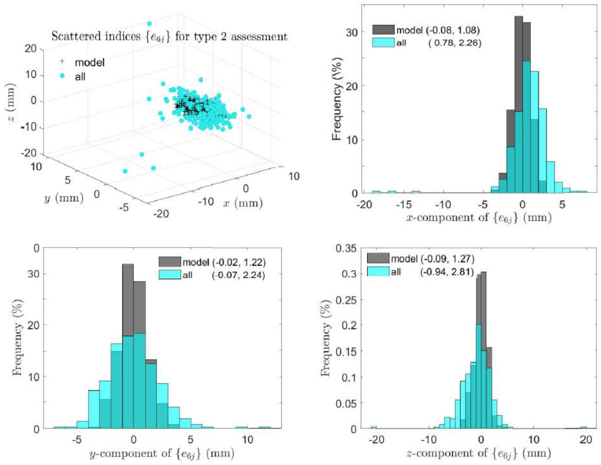

Fig S6. Type 2 assessment: Directional asymmetry of landmark pairs. Scattered points and histograms of asymmetry indices of $\{e_{6j}\}$ from the 359 perturbed symmetric skull and all real skulls, where in brackets are the means and standard deviations of components of $\{e_{6j}\}$.
